# Evaluation of an Automated Analysis Tool for Prostate Cancer Prediction Using Multiparametric Magnetic Resonance Imaging

**DOI:** 10.1371/journal.pone.0159803

**Published:** 2016-07-25

**Authors:** Matthias C. Roethke, Timur H. Kuru, Maya B. Mueller-Wolf, Erik Agterhuis, Christopher Edler, Markus Hohenfellner, Heinz-Peter Schlemmer, Boris A. Hadaschik

**Affiliations:** 1 Department of Radiology, German Cancer Research Center (DKFZ), Heidelberg, Germany; 2 Department of Urology, University Hospital of Cologne, Cologne, Germany; 3 Watson Medical bv, Den Ham, The Netherlands; 4 Department of Urology, University Hospital Heidelberg, Heidelberg, Germany; Chinese Academy of Sciences, CHINA

## Abstract

**Objective:**

To evaluate the diagnostic performance of an automated analysis tool for the assessment of prostate cancer based on multiparametric magnetic resonance imaging (mpMRI) of the prostate.

**Methods:**

A fully automated analysis tool was used for a retrospective analysis of mpMRI sets (T2-weighted, T1-weighted dynamic contrast-enhanced, and diffusion-weighted sequences). The software provided a malignancy prediction value for each image pixel, defined as Malignancy Attention Index (MAI) that can be depicted as a colour map overlay on the original images. The malignancy maps were compared to histopathology derived from a combination of MRI-targeted and systematic transperineal MRI/TRUS-fusion biopsies.

**Results:**

In total, mpMRI data of 45 patients were evaluated. With a sensitivity of 85.7% (with 95% CI of 65.4–95.0), a specificity of 87.5% (with 95% CI of 69.0–95.7) and a diagnostic accuracy of 86.7% (with 95% CI of 73.8–93.8) for detection of prostate cancer, the automated analysis results corresponded well with the reported diagnostic accuracies by human readers based on the PI-RADS system in the current literature.

**Conclusion:**

The study revealed comparable diagnostic accuracies for the detection of prostate cancer of a user-independent MAI-based automated analysis tool and PI-RADS-scoring-based human reader analysis of mpMRI. Thus, the analysis tool could serve as a detection support system for less experienced readers. The results of the study also suggest the potential of MAI-based analysis for advanced lesion assessments, such as cancer extent and staging prediction.

## Introduction

Prostate cancer is the most common cancer in Europe and the second most common cancer worldwide for men [1]. Therefore, reliable and early detection of prostate cancer has become an important priority in the field of urologic oncology.

Multiparametric magnetic resonance imaging (mpMRI) is emerging as the state-of-the-art method for providing diagnostic information on prostate cancer. MRI-based diagnostic information has been proven to be of vital importance as input to image-guided targeted biopsy methods that are deployed to seek pathologic confirmation [2]. MRI-targeted prostate biopsy detects more clinically significant prostate cancer than conventional systematic biopsy [3,4], and a change in clinical practice away from random sampling towards image-guided biopsies therefore seems attractive. Standards for the analysis of mpMRI images by human readers have recently emerged; the most widely accepted being those published by the European Society for Urogenital Radiology (ESUR) [5]. The ESUR guidelines also describe a standardized scoring system (PI-RADS) to characterize the malignancy expectation to be attributed to lesions identified [6].

Concomitantly to the development of human reader based qualitative analysis techniques, much effort has been spent on quantitative analysis of mpMRI data and especially on the relationship between quantifiable characteristics and detection of malignancy. This has led to the development of computer-aided diagnosis (CAD) systems that aim to facilitate human readers’ assessment of mpMRI prostate data. The first developed systems concentrated on specific areas of the prostate, mostly the peripheral zone [7,8], while more recent systems assessed the entire prostate [9,10]. The software evaluated in this study analyses, both, the peripheral and the central parts of the prostate.

The aim of the study was to evaluate the diagnostic accuracy of a novel software tool that autonomously analyses mpMRI images of the prostate and provides a malignancy assessment that is directly linked to prostate anatomy.

## Materials and Methods

### Patients

This retrospective study was performed on mpMRI data from a cohort of 45 patients that underwent mpMRI and subsequent MR/TRUS fusion biopsy of the prostate.

The study was conducted in concordance with the standards of the local ethics committee. Informed written consent was obtained from all patients for evaluation of the data. The cohort consisted of a, randomly selected, sub- group of patients previously accrued for a prospective study [11]. All patients had prior clinical suspicion of prostate cancer. None of the patients in the cohort under investigation had undergone prior prostate cancer therapy. Furthermore, patients with a prior diagnosis of insignificant prostate cancer and patients under active surveillance were not included into the study.

### Imaging technique

All images for the study were acquired with 3.0 Tesla MRI (Siemens Trio, Siemens Healthcare, Erlangen, Germany), using a standard multi-channel body coil and integrated spine phased-array coil. For each patient, three different sequences were used in the analysis, according to the following protocols:

- T2-weighted transversal images based on a high spatial resolution Turbo Spin Echo (TSE) sequence
- Diffusion-weighted transversal images based on a 2D-Echo Planar Imaging (EPI) sequence
- Dynamic Contrast Enhanced (DCE) transversal images based on a Time-resolved angiography With Interleaved Stochastic Trajectories (TWIST) sequence

The diffusion-weighted images (DWI) were acquired at seven different b-values, from which apparent diffusion coefficient (ADC) maps were computed. The DCE images were acquired at a temporal resolution of 9.9 s, using a body weight adjusted bolus of intravenously injected gadobutrol (0.1 mmol/kg body weight, Gadovist^®^, Bayer Healthcare, Leverkusen, Germany).

The detailed MR acquisition parameters are presented in Table 1.

**Table 1:** Parameter settings of deployed multiparametric MRI sequences.

Parameter	T2 TSE	Epi-2D	TWIST
TR (ms) / TE (ms)	5120/143	3100/52	4.42/2.2
Flip angle (°)	90	90	15
ETL length/epi-factor	12	96	-
Number of averages	4	5	-
b values	-	0,50,100,150,200,250,800	-
Slice thickness (mm)	3	3	1.5
FOV (mm)	300	280	400
Pixel size (mm x mm)	0.8 x 0.7	2.2 x 2.2	1.6 x 1.6
Acquisition time (min:s)	4:14	5:04	5:18

### Pathologic reference

The pathologic reference for this study was provided using a combination of an MRI-targeted and a systematic biopsy protocol that was implemented using a transperineal image-guided biopsy technique [12]. The technology that was used, documented the 3-dimensional position of every core taken allowing for accurate establishment of the relationship between pathologic findings and the specific anatomic sites, as seen in the MR-images, from which the biopsy cores were acquired. In addition to the targeted biopsy, all patients underwent a systematic transperineal saturation biopsy as a reference, which is known to correlate well with final pathology grading results [13,14].

Lesions for the targeted part of the protocol were identified by two board-certified radiologists, based on the PI-RADS scoring system. Consequential to the fact that the study was conducted retrospectively, when lesions identified by the software were not previously identified by the radiologists, they could only be confirmed or denied by the systematic part of the protocol and only as far as its coverage would include those lesions.

Gleason scores of 6 and higher were considered positive for prostate cancer. The average total number of biopsies taken per patient (i.e. targeted and systematic combined) was 24 (minimum 16, maximum 36).

### Automated malignancy analysis

For automated malignancy assessment, the evaluated software (Watson Elementary, Watson Medical, Den Ham, The Netherlands) computed a pixel-wise malignancy prediction value, defined as Malignancy Attention Index (MAI) from information contained in the T2W, DWI and DCE images. For calculation, several basic imaging features (see second step) correlating with prostate cancer [5,8,15] were quantified and compared against predefined prediction certainty levels. The malignancy prediction algorithm then further processed the quantified parameters, using a multivariate analysis method, to yield a single predictive value for each image pixel, i.e. MAI, minimizing the overall uncertainty level of this value. The MAI ranged between 0 and 1, with 0 indicating the lowest level of predicted malignancy and 1 the highest. Because the MAI was calculated for each image pixel, it could be mapped onto the MR images, for instance onto the T2W images, to illustrate the relationship with the prostate anatomy.

The automated analysis was performed in three steps:

1. Co-registration of DWI and DCE with T2W images
2. Computation of basic functional parameters
3. Computational analysis of image features and basic functional parameters to produce a malignancy prediction map

The co-registration step was necessary to enable accurate spatial correlation of relevant features from the different image sets. In the second step, basic functional parameters were computed from the DWI and DCE image data. For DWI, a pixel-based ADC value was calculated. For DCE, a set of pharmacokinetic parameters (K^trans^, V_e_ and k_ep_) related to a two compartment Tofts model was used [16]. Furthermore, normalized T2W data was generated using a rectangular computational volume of interest encompassing the prostate as closely as possible. The final step involved the actual malignancy prediction computation, which used output from the first and second step as input for a computational analysis of image features and basic functional parameters. This resulted in a pixel-wise MAI map.

**Fig 1:**
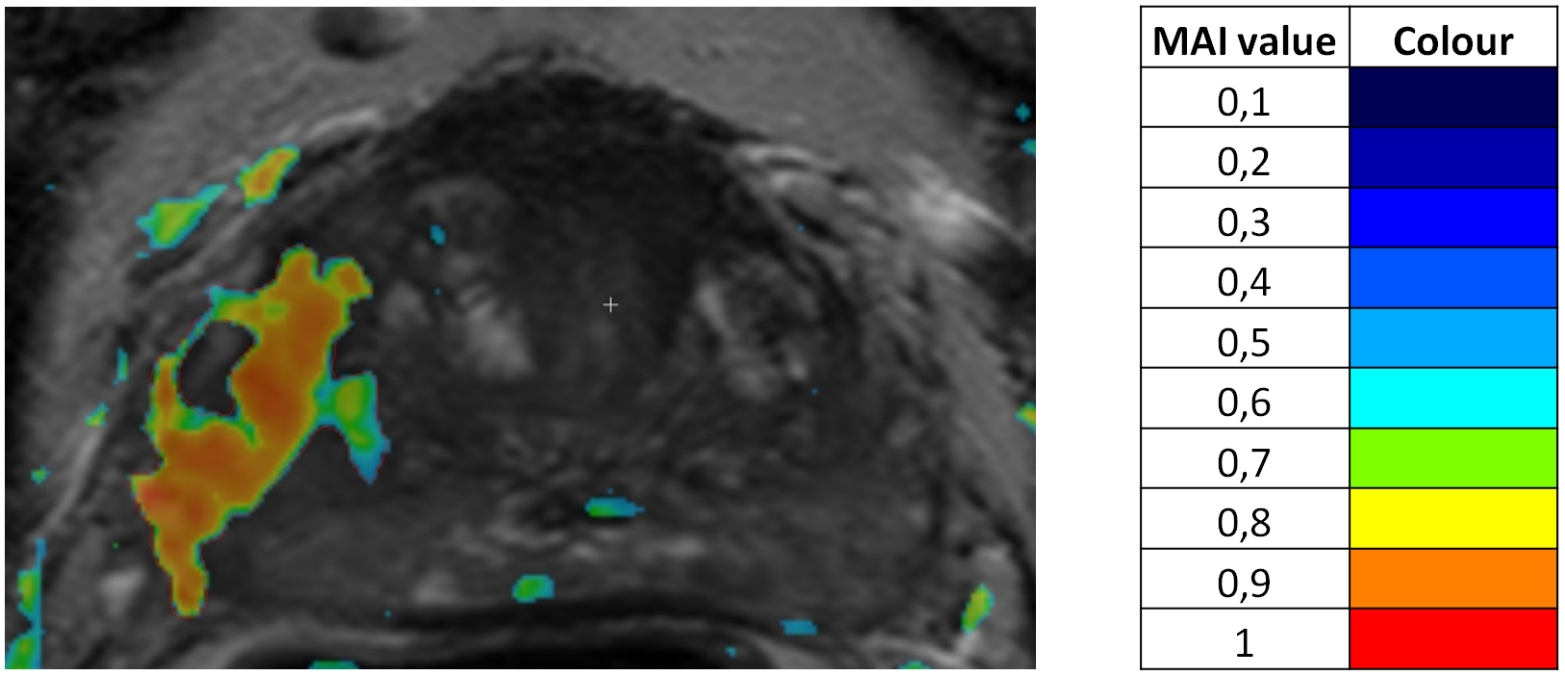
Example of a MAI map on a T2W image overlay.

Fig 1 shows an example of an MAI map in overlay on a T2W image, with red colour indicating high MAI values and colours towards blue indicating lower values, ending, at the low end, in transparency. Volumes characterized by elevated MAI values automatically constituted regions of interest (ROI), thus, no prior manual choice of ROI was required.

**Fig 2:**
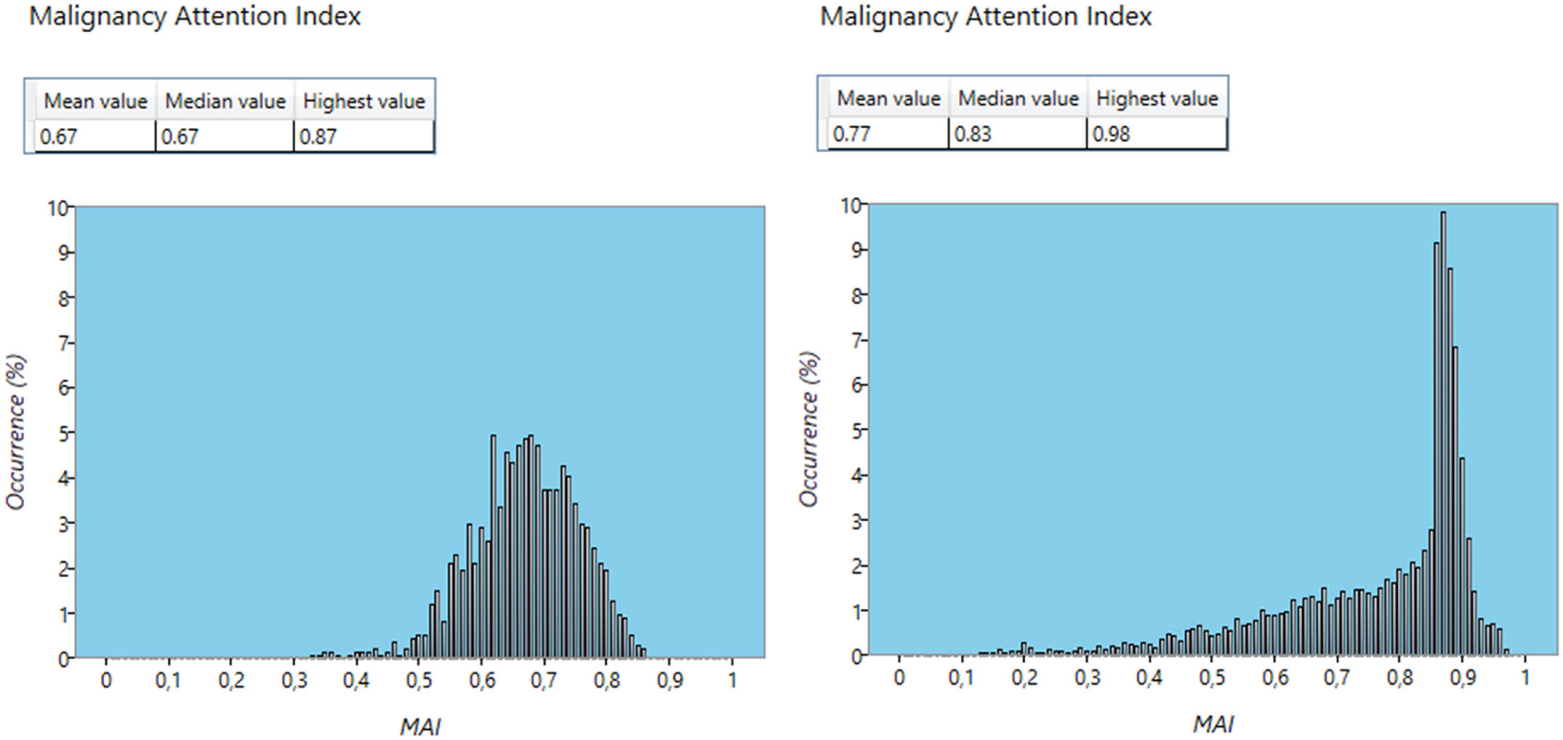
Examples of MAI profiles. Confirmed Gleason score 7a (3+4, left) and Gleason score 8 (4+4, right) lesions.

The software additionally generated histograms showing the MAI distribution over a predefined volume. These distributions could be interpreted as malignancy profiles. Fig 2 shows two examples of MAI profiles: one for a lesion with confirmed Gleason score 8 (4+4) and a second with confirmed Gleason score 7a (3+4).

The original features, as identified in the different image sets, are each represented by a scalar value, thus together forming a multi-dimensional feature space. Top-down, the MAI algorithm can be viewed as a complex, non-linear, mapping between this feature space and the one-dimensional, scalar, MAI space.

**Fig 3:**
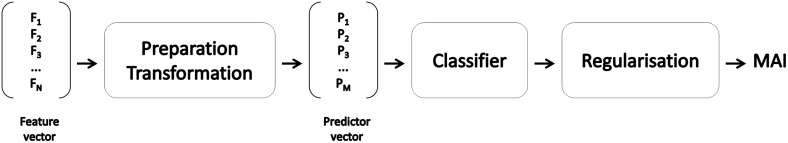
Overview of MAI algorithm steps.

The MAI algorithm achieves this mapping in three steps, as depicted in Fig 3. The first step, which we shall call the preparation transformation, involves projecting the raw feature vectors into another space of different dimension, which we shall call predictor space, in such a way that they become suited for the actual classification step. This latter step involves a linear classifier that maps predictor space onto a malignancy measure. Finally, a regularisation step is applied to create congruence with Gleason grade.

Each of the individual components of the feature vector has a particular classification error profile associated with it. For instance, very low or high ADC values can be associated with respectively the presence or absence of prostate cancer with a smaller margin of error than mid-range ADC values [8]. The central goal of multi-parametric analysis, irrespective of the method chosen, is to combine the information embedded in the individual features in such a way that the overall classification has a significantly lower error level than that provided by the individual features. See in relation to this point for instance the discussion of the improvement in detection accuracy when a formalized combined malignancy likelihood scoring method is used, compared to scoring on individual image sequences, as provided in the study by Roethke et al [11]. In fact, this is the predominant function of the first step of the algorithm, which re-arranges and combines the feature information into appropriate vectors in predictor space, such that the influence of high error components in the input is minimized and the influence of low error components is maximized. It also ensures that the second step can provide a consistent malignancy measure, i.e. a measure that consistently rises or diminishes in accordance with changes in individual features. The first step of the algorithm is mathematically equivalent to a kernel-based artificial neural network [17,18].

The main function of the second step is to provide the actual mapping onto a single scalar value. It is mathematically equivalent to the summation layer in some classic artificial neural network architectures, such as radial basis function (RBF) networks [19,20].

Although the output of the second step already provides a consistent malignancy measure, it is hard to compare it to generally accepted measures, such as Gleason grade. Therefore, in a third step, a mapping takes place to provide congruence with Gleason grade.

The algorithm was trained by means of a supervised learning process, making use of a separate dataset in which malignant volumes were clinically identified in advance. The dimensions of predictor space as well as the transformation parameters involved in the first step of the algorithm and the linear summation parameters of the second step were constructed dynamically by deploying an error-feedback mechanism.

First, the first two steps of the algorithm were trained only to provide the correct ordering of the input data set; i.e. in the correct order of pre-determined malignancy grade. Then, the third step was added to the training process to achieve congruence with Gleason grade. For this step, a generalised distance metric was used to quantify classification errors, while a constraint on the ordering achieved by the first training set was applied to maintain consistency.

### Image co-registration method

A vital precursor to the MAI calculation, and as such the first of the three steps comprising the automated malignancy analysis process, is an image co-registration procedure. It divides the two image data sets that are to be matched into a primary and a secondary set. The secondary set is first resampled to match the spatial resolution of the primary set. Subsequently, the algorithm deploys an iterative process to find an affine transformation that maps the secondary image onto the primary image in such a way that anatomical features in both images are satisfactorily overlaid. In each iteration, the quality of the match up to that point is determined by means of a mutual information metric [21]. Changes in the proposed transformation from one iteration to the next are governed by a conjugate gradient method [22]. The transformation involves translations, rotations and scaling, each pertaining to all 3 possible degrees of freedom, bringing the total number of degrees of freedom being optimized to 9. The starting point, or ‘initial guess’, for the transformation is calculated from the frame of reference and field of view information that is provided by the MRI scanner as described in the DICOM standard.

Once the images have been co-registered in the above manner, there exists a pixel-by-pixel correlation between them. This means that features from the individual images can then be combined into a set of feature vectors, associating each pixel with its own vector in feature space, which in turn is the input to the MAI algorithm.

### Image-guided biopsy

All biopsies were obtained by means of a transperineal approach, using a system (BiopSee^®^, Medcom, Darmstadt, Germany) [12] that allows co-registration of MRI with transrectal ultrasound images to provide live image guidance. The system features a mechanically fixed relationship between the ultrasound probe and the needle guidance mechanism to increase spatial accuracy. The system provides a high level of spatial accuracy, which was demonstrated previously [23].

In order to obtain a set of biopsies using this system, firstly, the MR images were adorned with information about the lesions’ positions and their respective dimensions. Next, on the MR images, the operator planned the exact target positions from which biopsy cores were retrieved. Then, the system co-registered the MR images and the biopsy plan with a 3D ultrasound volume acquired at the time of the biopsy. Finally, the system provided accurate live guidance of the biopsy needles towards the planned positions and recorded the actual positions from which the biopsy cores were obtained. This information could later be used to correlate pathology findings to anatomic locations, especially, as indicated by the original MRI-based lesion assessment.

### Analysis methods

The analysis was performed by projecting the biopsy core positions, which are known from the image-guided biopsy procedure, into the malignancy prediction maps. This way, the correlation between the predicted malignancy at each position and the actual pathology data of every single core could be assessed.

Each biopsy core was treated as a separate volume, thus, enabling the calculation of an MAI profile for each core. A core’s MAI profile was considered predictive of a positive pathologic assessment if a fixed set of numeric features exceeded a set of corresponding threshold values.

As the analysis algorithm was designed to exhibit a linear correlation of the MAI with the Gleason score [24], the first threshold feature was chosen to be the highest MAI value present in the profile. The threshold for this feature was set at a value of 0.6, corresponding to the lowest Gleason score associated with prostate cancer on biopsy-specimen (i.e. Gleason score 6). Furthermore, a threshold was put on the mean MAI value and varied. By doing so, we were able to assess the influence of MAI distribution shape on diagnostic accuracy. With higher MAI thresholds expected to correspond to higher Gleason score thresholds, the detection sensitivity was expected to fall and the specificity to rise at higher MAI thresholds. This hypothesis was then tested by ROC analysis and Youden J statistics (Youden index).

A predicted malignant lesion was considered true positive if at least one biopsy core taken from that lesion was found positive in pathology assessment, as well as, in the MAI-based analysis. If no biopsy cores taken from a predicted malignant lesion were found positive in pathology assessment, it was considered disproved. If no biopsy cores were taken from a predicted malignant lesion, it was left out of the analysis.

As the scoring of individual lesions in a single patient can be expected to be correlated, an individual patient scoring was adopted to ensure uncorrelated measurements. If, for a particular patient, at least one predicted malignant lesion was confirmed this was recorded as a ‘true positive’ score. If no lesions were predicted and none were found, we noted a ‘true negative’ score. If no lesions were predicted, yet malignancies were confirmed by pathology, this was marked as a ‘false negative’. By inference, all other scores were noted as ‘false positives’.

To measure the diagnostic accuracy of the MAI at different threshold settings, we computed sensitivity and specificity values at a 95% Wilson confidence interval, respectively. All statistical assessments of population differences were calculated using a Wilcoxon rank-sum test with a two-tailed *P* value threshold of 0.05.

## Results

### Patients

The 45 patients had a median age of 66 years (range, 49 to 77). The median baseline serum PSA level of these patients was 7.86 ng/ml (range, 1.75 to 39.2 ng/ml). Although the median PSA level of patients found positive for prostate cancer (9.15 ng/ml) was higher than that of patients who were tested negative for prostate cancer (7.3 ng/ml), statistically the difference only tended towards significance (P = 0.063). The median estimated prostate volume was 45 cm^3^ (range 22 to 96 cm^3^). The difference in prostate volume of patients finally confirmed with having prostate cancer and those without was not significant.

### Pathology

In total, 1102 individual biopsy cores were analyzed. Of those cores, 1026 were found negative for prostate cancer, whereas, 76 cores were found positive. The positive pathology findings were attributed to 21 different patients, yielding a prostate cancer detection rate of 46.7%. The median Gleason score for patients found positive for prostate cancer was 7 (3+4). The lowest Gleason score deemed positive for prostate cancer was 6.

### ROC analysis

**Fig 4:**
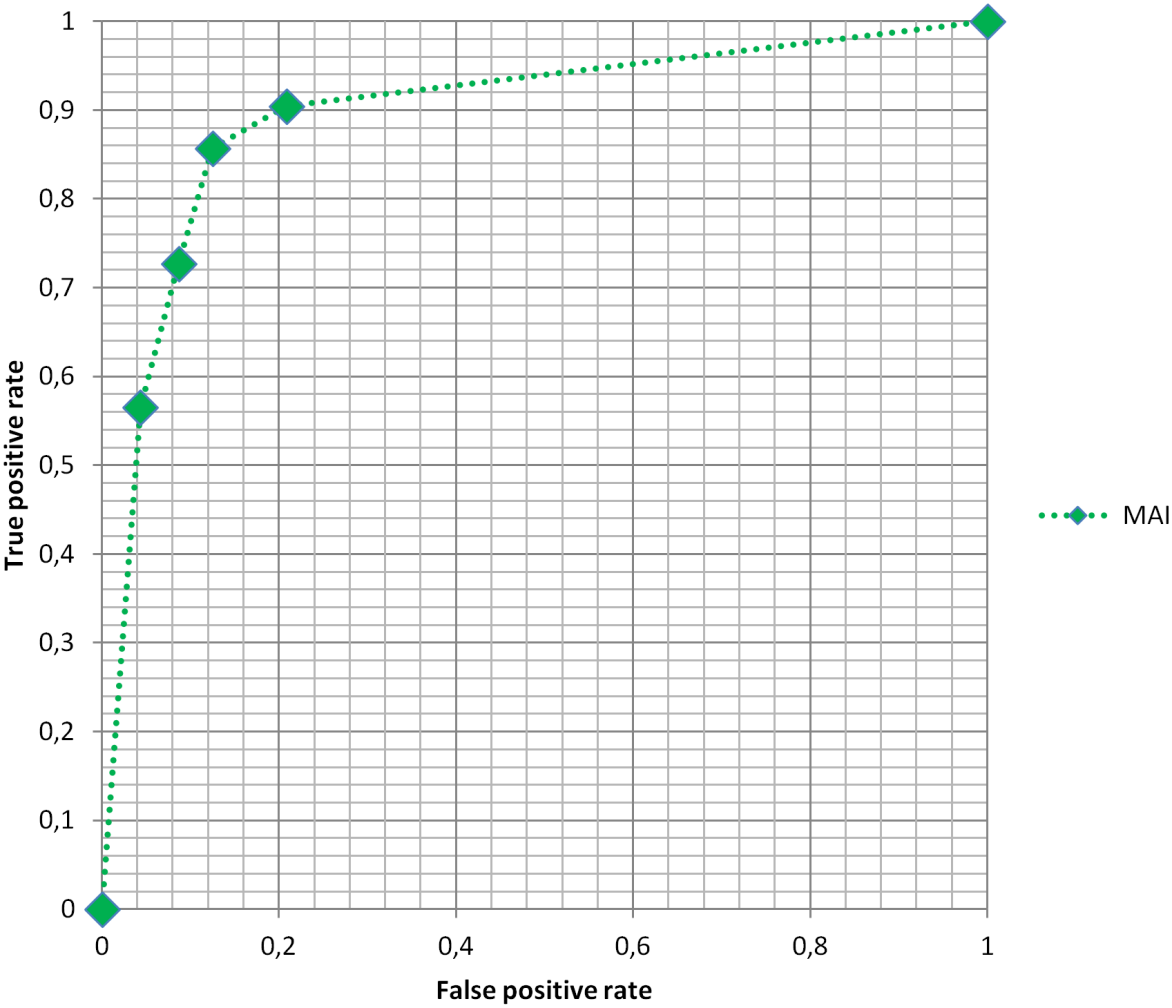
ROC plot of MAI diagnostic accuracy.

By means of varying the threshold on the mean MAI value, while keeping the threshold on the highest MAI value constant (i.e. 0.6), we determined a set of sensitivity and specificity values. Fig 4 shows the resulting ROC plot (see Table 2 for exact values and confidence intervals). More information is available as support download file named S1 Data.

**Table 2:** Sensitivity, specificity and Youden index of MAI-based prostate cancer detection at different mean MAI threshold values.

Mean MAI threshold	0.15	0.2 (optimum)	0.3	0.4
Sensitivity	90.48% (71.09–97.35)	85.71% (65.36–95.02)	72.73% (51.35–87.08)	56.52% (35.99–75.03)
Specificity	79.17% (59.53–90.76)	87.50% (69–95.66)	91.30% (73.69–97.52)	95.65% (79.50–99.21)
Detection accuracy	84.44% (71.22–92.25)	86.67% (73.83–93.75)	82.22% (68.67–90.71)	76.09% (61.90–86.17)
Youden index	69.64% (30.62–88.10)	73.21% (34.36–90.67)	64.03% (25.04–84.6)	52.17% (15.49–74.24)

The Youden-selected optimum computed mean MAI threshold was found to be 0.2, which corresponds to an 85.7% sensitivity (with 95% CI of 65.4–95.0) combined with an 87.5% specificity (with 95% CI of 69.0–95.7) and a diagnostic accuracy of 86.7% (with 95% CI of 73.8–93.8) in the detection of prostate cancer with Gleason score > = 6. The Youden index for this optimum was 73.2% (with 95% CI of 34.36–90.67). The area under the curve (AUC) was 0.90 (with 95% CI of 0.66–0.98).

## Discussion

### General observations

The results of the present study demonstrated diagnostic accuracy of an automated analysis of mpMR images, by means of an MAI computation, that are comparable to the results of human readers in prior validation studies of the ESUR system.

There was a clear optimum in diagnostic accuracy associated with a set of threshold values pertaining to easily computed features of MAI profiles. When varying the threshold on the mean MAI value, the diagnostic accuracy varied according to expectation: at higher threshold values, the detection sensitivity decreased, whereas, the specificity increased.

Other studies have shown similar behaviour for PI-RADS-based lesion assessment and Gleason score histopathology-based lesion assessment under varying scoring thresholds [6,11,25,26]. The results published by Roethke et al. [11] and Schimmöller et al. [25] showed the same trend in sensitivity and specificity under varying threshold values, yet differ considerably in terms of specificity at lower threshold values (73% [11] versus 41.7% [25] at a PI-RADS threshold value of 9). Nonetheless, although at the lower threshold the specificity reported by Schimmöller et al. is lower than that reported by Roethke et al., the sensitivity reported is slightly higher. Also, the resulting point on the ROC curve coincides quite well with the results of the other studies. A similar observation could be made regarding the results reported by Hamoen et al. [6]. In this case, the reported sensitivities and specificities at different threshold values were closer together than the values reported by Roethke [11] and Schimmöller [25] et al., yet they were again close to the ROC trend lines for those studies and to that of the current study. The authors therefore believe that the observed differences are possibly suggestive of differences in human scoring preferences. All studies showed a quick saturation of the ROC curves at lower threshold values, which was suggestive of a limitation of the amount of useable information in the MR-images. The congruence in behaviour with other, widely accepted, methods for malignancy assessment [5] indicated that MAI based analysis assesses the same underlying pathologic correlation as the other methods.

### Comparison to human reader diagnostic accuracy

**Fig 5:**
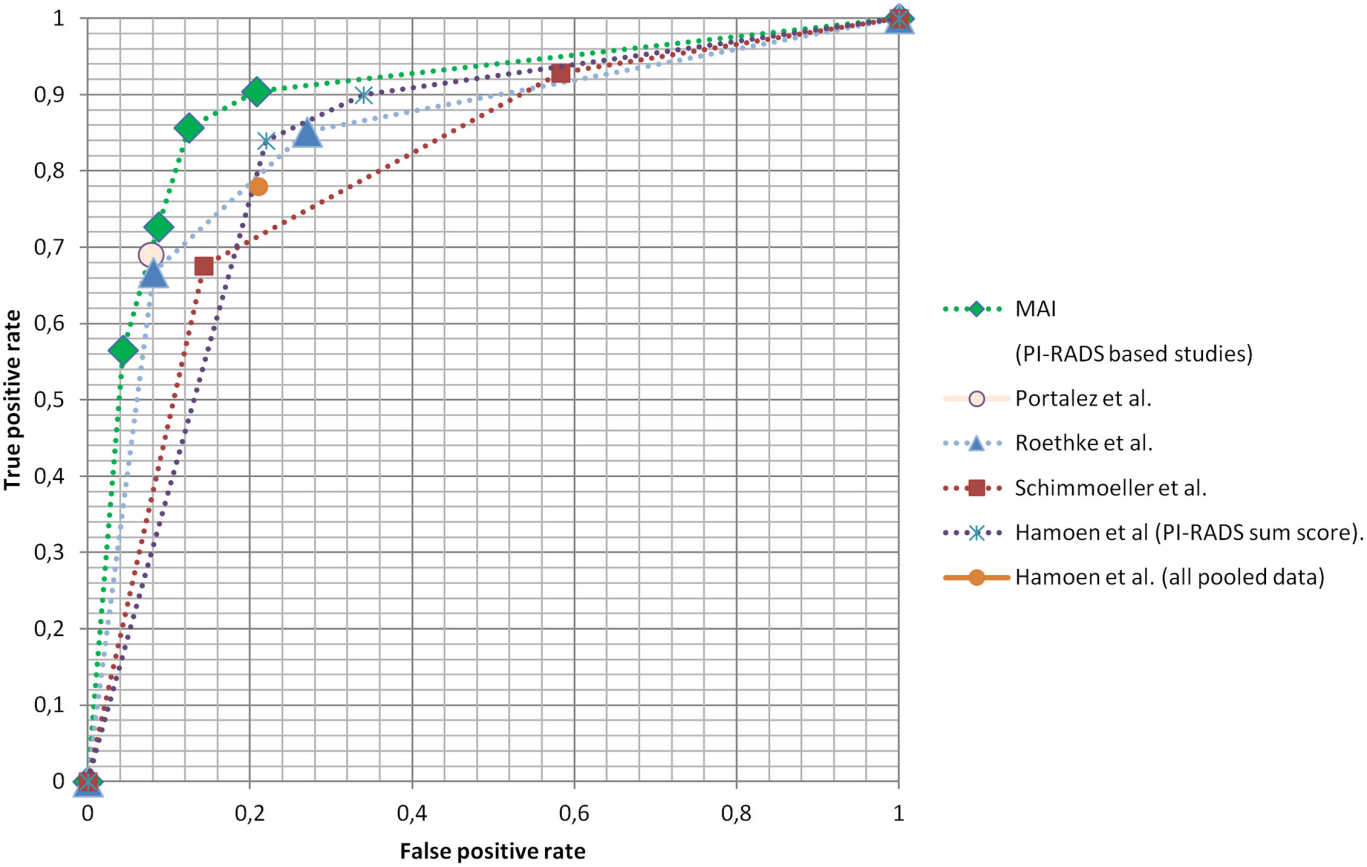
Comparison of computed MAI diagnostic accuracy with that of human readers based on PI-RADS scoring.

We compared the diagnostic accuracy of the computed MAI to previously reported diagnostic accuracies of human readers. Since the patient cohort for this study was a subset taken from the cohort used in an earlier study by Roethke et al. [11] into the diagnostic accuracy of human readers, scoring according to the PI-RADS system, we consider the comparison with this study to be the most accurate and hence made it our primary reference. Fig 5 shows a comparison between the diagnostic accuracy based on MAI and the ROC of the human reader diagnosis based on PI-RADS scoring at PI-RADS threshold values 9 and 10 as reported by Roethke et al. [11]. Table 3 shows a numeric comparison. The values reported in the reference study fall well within the confidence interval reported in this study for the computed MAI.

**Table 3:** Comparison of the diagnostic accuracy found in the present study with results reported on reference studies.

	Present study	Roethke et al.		Schimmöller et al.		Portalez et al.	Hamoen et al.		
	MAI (optimum)	PI-RADS		PI-RADS		PI-RADS	PI-RADS		Pooled data, no further selection
		≥ 9	≥ 10	≥ 9	≥ 10	≥ 9	≥ 9	≥ 10	
Sensitivity (%)	85.71 (65.36–95.02)	85.2 (66.3–95.8)	66.7 (46.0–83.5)	92.9	85.7	69.1 (56.7–79.8)	90 (75–96)	84 (74–90)	78 (70–84)
Specificity (%)	87.50 (69–95.66)	73.0 (55.9–86.2)	91.9 (78.1–98.3)	41.7	67.6	92.2 (89.2–94.5)	66 (38–87)	78 (62–89)	79 (68–86)
Youden index (%)	73.21 (34.36–90.67)	58.2 (38.6–77.8)	58.6 (38.7–78.4)	-	-	-	-	-	
AUC	0.90 (0.66–0.98)	0.848 (0.743–0.953)		-		0.873	-	-	

The trend line drawn through the values measured in the reference study provides further indication of the close relationship between diagnostic accuracy based on MAI computation and that of expert human readers.

In their study on inter-reader agreement of the ESUR (PI-RADS) scoring system using in-bore MRI-guided biopsies as reference standard, Schimmöller et al. also reported diagnostic accuracies at PI-RADS threshold values of 9 and 10 [25]. The result at threshold value 10 falls within the confidence interval of the computed MAI diagnostic accuracy. When compared to the result at threshold value 9, the optimum computed MAI provides a higher diagnostic accuracy (Fig 5 and Table 3).

In their validation study of the ESUR scoring system, Portalez et al. reported a diagnostic accuracy at a Youden-selected PI-RADS score threshold value of 9 [26]. Although this threshold corresponded to the Youden-selected threshold in the other two reference studies, the diagnostic accuracy reported in the latter study corresponded mostly with those reported in the other two studies at a threshold value of 10. However, this result also fell within the confidence interval of the optimum computed MAI diagnostic accuracy.

Finally, in their meta-analysis of reported detection capabilities of mpMRI in combination with the PI-RADS scoring system, Hamoen et al. [6] reported prostate cancer detection sensitivities and specificities of 90% (95% CI 75%–96%) and 66% (95% CI 38%–87%) for a PI-RADS score threshold of 9, and 84% (95% CI 74%–90%) and 78% (95% CI 62%–89%) for a threshold of 10, for pooled studies that deployed PI-RADS sum-scores. Furthermore, they found a prostate cancer detection sensitivity of 78% (95% CI 70%-84%) and a specificity of 79% (95% CI 68%-86%) for pooled studies, irrespective of further selection criteria. Again, these numbers were in line with the outcome of the current study.

### Granularity

An important difference between the MAI model and the PI-RADS model was that the latter yielded a fixed number of discrete scores, whereas, the MAI model was continuous. The ROC analysis showed that, as a consequence, the MAI model allowed for a higher granularity in optimization. In their study, Roethke et al. [11] observed that choosing a PI-RADS threshold was guided by a weighing of sensitivity versus specificity as either choice yielded comparable Youden J statistics (see Table 3). Due to its continuous nature, the MAI model did not require such decision-making. This led to a higher achievable Youden index and the avoidance of low threshold values leading to only a slight improvement in sensitivity at the cost of a high loss in specificity.

### Factors of influence on measured diagnostic accuracy

Several effects influenced the measured diagnostic accuracies. First of all, the fact that the present study was performed in a retrospective setting meant that only lesions that were originally predicted by expert human readers were indeed targeted. The software predicted a total of 3 lesions in 2 different patients that were considered clinically relevant (mean and highest lesion MAI > 0.6 and volume > = 0.5ml) yet were not biopsied, while no positive biopsy cores were found in other locations. This amounted to 4.4% of the patient cohort. According to this study’s authors, however, this did not significantly influence the value of the outcome of the study for the purpose of targeted biopsy planning. At worst, the implication could have been a small increase of the false positive rate, which would have resulted in a specificity of 79.17% at an unchanged sensitivity of 85.71% and a Youden index of 64.88%. Altogether, this would have been still well within the confidence intervals of the optimum accuracies found in the reference studies. For other purposes, though, such as focal therapy planning, the authors acknowledge that prospective data is needed to settle the issue of negative predictive strength.

A second phenomenon that may have affected measured diagnostic accuracy was limited effective biopsy core length. Several studies [27,28] have shown a clear relationship between detection rate and effective core length, being the length actually acquired versus the maximum potential length of a core based on needle configuration. This was expected to be of influence on the presently reported results. Particularly, this may have been the case at lower mean MAI threshold values, as one of the possible reasons for a core exhibiting a low mean MAI was its lesion overlapping with only a small part of the core. The probability of not achieving pathologic confirmation due to limited effective core length was higher when only a small portion of the planned core overlapped with the lesion, compared to when a larger portion overlapped. In summary, when using a biopsy-based pathology reference, the number of reported false positives will increase at lower mean MAI thresholds due to limited effective core length effects. Because performing a biopsy is the de-facto standard method to confirm or deny suspected prostate cancer, the outcome of the present study, including limited core length effects, reflects the practically achievable overall diagnostic accuracy in a pre-therapeutic setting. Analysis of post-prostatectomy whole-mount specimen may contribute to an incremental better accuracy. Furthermore, the authors conclude that any differences between human reader diagnostic accuracy and computed MAI based diagnostic accuracy cannot be attributed to limited effective core length effects as both methods are affected equally by this phenomenon.

Concerning influences on diagnostic accuracy as described above, in general, lower threshold values lead to an increase in detection sensitivity. However, any lesion assessment method exhibits practical limitations that lead to an associated ROC curve saturating at a maximum sensitivity level below the theoretical maximum value of 1. In the present study, no biopsy cores were found to have a maximum MAI score of minimally 0.6 combined with a mean MAI score smaller than 0.15. Hence, the sensitivity saturation level for this particular study setup was found to be 90.48% (see also Table 2).

### Practical optimization of needle placement

The presented analysis method and results could easily be converted into practical guidelines for planning optimal image guided biopsies based on MAI analysis. Since MAI values are available for each image pixel, they could be colour coded and projected onto the T2W image set for anatomic reference.

Since the colour coding has a fixed relationship with MAI value, it is straightforward to optimize for detection accuracy in planning biopsy cores. An optimal biopsy plan enables the retrieval of cores with the highest volume of the highest possible MAI values. The current study showed that results comparable to planning by experienced human readers could be achieved by planning cores in areas where the colour code indicated an MAI above a threshold value of 0.6. However, it was not necessary to explicitly keep track of the mean MAI in biopsy core planning when deploying the aforementioned optimization strategy. It would suffice to try and plan to obtain as much volume as possible of the highest possible MAI values to be included in each biopsy. Such a strategy automatically leads to inclusion of cores that fall above the optimal mean MAI threshold reported in this study (0.2), if such cores can be planned practically. Any additionally planned cores with a mean MAI lower than the optimal reported threshold could only lead to a slightly increased sensitivity (up to the anticipated saturation value) at the cost of an increased number of negative pathology results.

### Limitations and recommendations for future research

The authors recognize that the limited number of patients included and the single-institutional setting are limitations of the current study. It is therefore recommended that more research is performed in a multi-institutional setting. Furthermore, the authors recommend that future studies be conducted in a prospective setting.

Earlier in the year 2015, the PI-RADS scoring system was updated to version 2. To date, few experiences with this system exist due to the recentness of this update. All comparisons with human reader studies in this study pertained to PI-RADS scoring system version 1.

## Conclusion

The study revealed comparable diagnostic accuracies for the detection of prostate cancer of a user-independent MAI-based automated analysis tool and PI-RADS scoring-based human reader analysis of mpMRI. Thus, the analysis tool could serve as a detection support system for less experienced readers. The results of the study fascilitate a straightforward biopsy planning strategy to yield optimal diagnostic accuracy based on computed MAI maps. The results also suggest future potential of computer-based analysis for advanced lesion assessments, such as cancer extent and staging prediction.

## Supporting Information

S1 Data (XLSX)

## References

[1] Ferlay J, Soerjomataram I, Ervik M. Cancer Incidence and Mortality Worldwide: IARC CancerBase No. 11. Lyon: Globocan (http://globocan.iarc.fr); 2012 Available: http://globocan.iarc.fr.

[2] Pokorny MR, Rooij de M, Duncan ESFH, Parkinson R, Barentsz JO, Thompson LC. Prospective Study of Diagnostic Accuracy Comparing Prostate Cancer Detection by Transrectal Ultrasound–Guided Biopsy Versus Magnetic Resonance (MR) Imaging with Subsequent MR-guided Biopsy in Men Without Previous Prostate Biopsies. European Urology. 2014; 66: 22–29. 10.1016/j.eururo.2014.03.002 24666839

[3] Siddiqui M, Rais-Bahram S, Turkbey B, George AK, Rothwax J, Shakir N, et al Comparison of MR/ultrasound fusion-guided biopsy with ultrasound-guided biopsy for the diagnosis of prostate cancer. JAMA. 2015; 313(4): 390–397. 10.1001/jama.2014.17942 25626035 PMC4572575

[4] Valerio M, Donaldson I, Emberton M, Ehdaie B, Hadaschik BA, Marks LS, M P, et al Detection of Clinically Significant Prostate Cancer Using Magnetic Resonance Imaging-Ultrasound Fusion Targeted Biopsy: A Systematic Review. Eur Urol. 2015; 68(1): 8–19. 10.1016/j.eururo.2014.10.026 25454618

[5] Barentsz JO, Richenberg J, Clements R, Choyke P, Verma S, Villeirs G, et al ESUR prostate MR guidelines 2012. Eur Radiol. 2012. 10.1007/s00330-011-2377-y PMC3297750 22322308

[6] Hamoen EHJ, Rooij Md, Witjes JA, Barentsz JO, Rosvers MR. Use of the Prostate Imaging Reporting and Data System (PI-RADS) for Prostate Cancer Detection with Multiparametric Magnetic Resonance Imaging: A Diagnostic Meta-analysis. Eur Urol. 2015; 67: 1112–1121. 10.1016/j.eururo.2014.10.033 25466942

[7] Vos PC, Hambrock T, Barentsz JO, Huisman HJ. Computer assisted analysis of peripheral zone prostate lesions using t2-weighted and dynamic contrast enhanced t1-weighted MRI. Phys. Med. Biol. 2010; 55(6): p. 1719 10.1088/0031-9155/55/6/012 20197602

[8] Shah V, Turkbey B, Mani H, Pang Y, Pohida T, Merina M, et al Decision support system for localizing prostate cancer based on multiparametric magnetic resonance imaging. Med. Phys. 2012; 39(7): 4093–4103. 10.1118/1.4722753 22830742 PMC3390048

[9] Hambrock T, Vos PC, Hulbergen-van de Kaa CA, B J.O., Huisman HJ. Prostate cancer: computer-aided diagnosis with multiparametric 3-T MR imaging-effect on observer performance. Radiology. 2013; 266: 521–530. 10.1148/radiol.12111634 23204542

[10] Tiwari P, Kurhanewicz J, Madabhushi A. Multi-kernel graph embedding for detection, Gleason grading of prostate cancer via MRI/MRS. Medical Image Analysis. 2013; 17(2): 219–235. 10.1016/j.media.2012.10.004 23294985 PMC3708492

[11] Roethke MC, Kuru TH, Schultze S, Tichy D, Kopp-Schneider A, Fenchel M, et al Evaluation of the ESUR PI-RADS scoring system for multiparametric MRI of the prostate with targeted MR/TRUS fusion-guided biopsy at 3.0 Tesla. Eur Radiol. 2013. 10.1007/s00330-013-3017-5 24196383

[12] Hadaschik BA, Kuru TH, Tulea C, Rieker P, Popeneciu IV, Simpfendörfer T, et al A novel stereotactic prostate biopsy system integrating pre-interventional magnetic resonance imaging and live ultrasound fusion. J. Urol. 2011; 186: 2214–2220. 10.1016/j.juro.2011.07.102 22014798

[13] Thompson JE, Moses D, Shnier R, Brenner P, Delprado W, Ponsky L, et al Multiparametric Magnetic Resonance Imaging Guided Diagnostic Biopsy Detects Significant Prostate Cancer and could Reduce Unnecessary Biopsies and Over Detection: A Prospective Study. J. Urol. 2014; 192(1): 67–74. 10.1016/j.juro.2014.01.014 24518762

[14] Lecornet E, Ahmed HU, Moore CM, Nevouw P, Barrat D, Hawkes D, et al The accuracy of different biopsy strategies for the detection of clinically important prostate cancer: a computer simulation. J. Urol. 2012; 188(3): 974–980. 10.1016/j.juro.2012.04.104 22819118

[15] Vos PC, Hambrock T, Hulsbergen-van de Kaa CA, Fütterer JJ, Barentsz JO, Huisman HJ. Computerized analysis of prostate lesions in the peripheral zone using dynamic contrast enhanced MRI. Med. Phys. 2008; 25(4): 621–630. 10.1118/1.2836419 18404925

[16] Tofts PS, Brix G, Buckley DL, Evelhoch JL, Henderson E, Knopp MV, et al Estimating Kinetic Parameters From Dynamic Contrast-Enhanced T1-Weighted MRI of a Diffusable Tracer: Standardized Quantities and Symbols. Journal Of Magnetic Resonance Imaging. 1999; 10: 223–232. 10508281 10.1002/(sici)1522-2586(199909)10:3<223::aid-jmri2>3.0.co;2-s

[17] Aizerman MA, Braverman EM, Rozoner LI. Theoretical foundations of the potential function method in pattern recognition learning. Automation and Remote Control. 1964; 25: 821–837.

[18] Hofmann T, Scholkopf B, Smola AJ. Kernel Methods in Machine Learning; 2008.

[19] Broomhead DS, Lowe D. Radial basis functions, multi-variable functional interpolation and adaptive networks. Royal Signals and Radar Establishment; 1988. Report No.: RSRE Memorandum No. 4148.

[20] Broomhead DS, Lowe D. Multivariable functional interpolation and adaptive networks. Complex Systems. 1988; 2: 321–355.

[21] Wells WM, Viola P, Atsumi H, Nakajima S, Kikinis R. Multi-modal volume registration by maximization of mutual information. Medical Image Analysis. 1996; 1(1): 35–51. 9873920 10.1016/s1361-8415(01)80004-9

[22] Hestenes MR, Stiefel E. Methods of Conjugate Gradients for Solving Linear Systems. Journal of Research of the National Bureau of Standards. 1952; 49(6): 409–436.

[23] Kuru TH, Roethke M, Popeneciu V, Teber D, Pahernik S, Zogal P, et al Phantom study of a novel stereotactic prostate biopsy system integrating preinterventional magnetic resonance imaging and live ultrasonography fusion. J. Endouroll. 2012; 26: 807–813. 10.1089/end.2011.0609 22283184

[24] Epstein JI, Allsbrook WC, Amin MB, Egevad LL. The 2005 International Society of Urological Pathology (ISUP) Consensus Conference on Gleason Grading of Prostatic Carcinoma. Am. J. Surg. Pathol. 2005; 29(9): 1228–1242. 16096414 10.1097/01.pas.0000173646.99337.b1

[25] Schimmöller L, Quentin M, Arsov C, Lanzman RS, Hiester A, Rabenalt R, et al Inter-reader agreement of the ESUR score for prostate MRI using in-bore MRI-guided biopsies as the reference standard. Eur Radiol. 2013; 23: 3185–3190. 10.1007/s00330-013-2922-y 23756958

[26] Portalez D, Mozer P, Cornud F, Renard-Penna R, Misrai V, Thoulousan M, et al Validation of the European Society of Urogenital Radiology Scoring System for Prostate Cancer Diagnosis on Multiparametric Magnetic Resonance Imaging in a Cohort of Repeat Biopsy Patients. European Urology. 2012; 62: 986–996. 10.1016/j.eururo.2012.06.044 22819387

[27] Kwast THvd, Lopes C, Santonja C, Pihl CG, Neetens I, Martikainen P, et al Guidelines for processing and reporting of prostatic needle biopsies. J. Clin. Pathol. 2003; 56(5): 336–340. 12719451 10.1136/jcp.56.5.336 PMC1769959

[28] Iczkowski KA, Casella G, Seppala RJ, Jones GL, Mishler BA, Qian J, et al Needle core length in sextant biopsy influences prostate cancer detection rate. Urology. 2002; 59(5): 698–703. 11992843 10.1016/s0090-4295(02)01515-7

